# Folding pathway of an Ig domain is conserved on and off the ribosome

**DOI:** 10.1073/pnas.1810523115

**Published:** 2018-11-09

**Authors:** Pengfei Tian, Annette Steward, Renuka Kudva, Ting Su, Patrick J. Shilling, Adrian A. Nickson, Jeffrey J. Hollins, Roland Beckmann, Gunnar von Heijne, Jane Clarke, Robert B. Best

**Affiliations:** ^a^Laboratory of Chemical Physics, National Institute of Diabetes and Digestive and Kidney Diseases, National Institutes of Health, Bethesda, MD 20892;; ^b^Department of Chemistry, University of Cambridge, Cambridge CB2 1EW, United Kingdom;; ^c^Department of Biochemistry and Biophysics, Stockholm University, SE-10691 Stockholm, Sweden;; ^d^Gene Center, Department of Biochemistry, Ludwig Maximilian University of Munich, 81377 Munich, Germany;; ^e^Center for Integrated Protein Science Munich, Ludwig Maximilian University of Munich, 81377 Munich, Germany;; ^f^Science for Life Laboratory, Stockholm University, SE-171 21 Solna, Sweden

**Keywords:** arrest peptide, fraction folded, mechanical force, molecular simulation, kinetic model

## Abstract

Proteins that fold cotranslationally may do so in a restricted configurational space, due to the volume occupied by the ribosome. How does this environment, coupled with the close proximity of the ribosome, affect the folding pathway of a protein? Previous studies have shown that the cotranslational folding process for many proteins, including small, single domains, is directly affected by the ribosome. Here, we investigate the cotranslational folding of an all-β Ig domain, titin I27. Using an arrest peptide-based assay and structural studies by cryo-EM, we show that I27 folds in the mouth of the ribosome exit tunnel. Simulations that use a kinetic model for the force dependence of escape from arrest accurately predict the fraction of folded protein as a function of length. We used these simulations to probe the folding pathway on and off the ribosome. Our simulations—which also reproduce experiments on mutant forms of I27—show that I27 folds, while still sequestered in the mouth of the ribosome exit tunnel, by essentially the same pathway as free I27, with only subtle shifts of critical contacts from the C to the N terminus.

## Significance

Most proteins need to fold into a specific 3D structure to function. The mechanism by which isolated proteins fold has been thoroughly studied by experiment and theory. However, in the cell proteins do not fold in isolation but are synthesized as linear chains by the ribosome during translation. It is therefore natural to ask at which point during synthesis proteins fold, and whether this differs from the folding of isolated protein molecules. By studying folding of a well-characterized protein domain, titin I27, stalled at different points during translation, we show that it already folds in the mouth of the ribosome exit tunnel and that the mechanism is almost identical to that of the isolated protein.

To what extent is the cotranslational folding pathway of a protein influenced by the presence of the ribosome and by the vectorial emergence of the polypeptide chain during translation? Recent studies have shown that small proteins can fold inside the ribosome exit tunnel (e.g., the small zinc-finger domain ADR1a) (1), while other proteins can fold at the mouth of the tunnel (e.g., the three-helix-bundle spectrin domains) (2); however, some proteins may be simply too large to fold within the confines of the ribosome (e.g., DHFR) (3). The nature of cotranslational protein folding is determined by a number of biophysical factors, including the folding properties of the isolated protein (4–9), together with the effects the ribosome itself may have on the folding process (10–16). Due to the spatial constraints imposed upon the nascent chain by the confines of the tunnel, and effects due to the close proximity of the ribosome itself, the ribosome has been shown to influence directly the cotranslational folding of small proteins and single domains: The stability of folded or partly folded states may be reduced when folding occurs close to, or within the confines of, the ribosome (17, 18); the folding kinetics are expected to be correspondingly altered, with the rate of folding likely to be decreased and the unfolding rate increased, in close proximity to the ribosome (18). Interactions of the folded state or nascent polypeptide with the ribosome may also be either stabilizing or destabilizing (19, 20). Since translation is vectorial in nature, it is possible that when proteins fold cotranslationally they fold via different pathways than those used when proteins fold outside the ribosome, or when isolated proteins fold in vitro (2, 11, 21–24). However, addressing these issues is challenging, because standard protein folding methods are not directly applicable to cotranslational folding.

The folding of the protein close to the ribosome generates a pulling force on the nascent chain. This force has been probed by single-molecule (25) as well as arrest peptide (AP) experiments (1–3). In this work, we use such AP-based cotranslational force-measurement experiments, simulations, and structural studies to investigate how the ribosome affects the folding of titin I27, a small all-β Ig domain with a complex Greek-key fold; the stability, kinetics, and folding pathway of I27 have been extensively characterized in previous studies of the isolated domain (26, 27). In this study we investigate whether I27 can begin to fold in the confines of the ribosome and if the folding pathway observed in the isolated domain is conserved during cotranslational folding. Results from all three techniques show that I27 folds in the mouth of the ribosome exit tunnel; our simulations correctly capture the onset of folding in I27 and three mutant variants, allowing us to predict how destabilization of regions that fold early and late in the isolated domain affect folding on the ribosome. Our simulations further show that the folding pathway of I27 is largely unaffected by the presence of the ribosome, except for small but significant changes observed for contacts near the N and C termini.

## Results

### I27 Folds Close to the Ribosome.

**Fig. 1.**
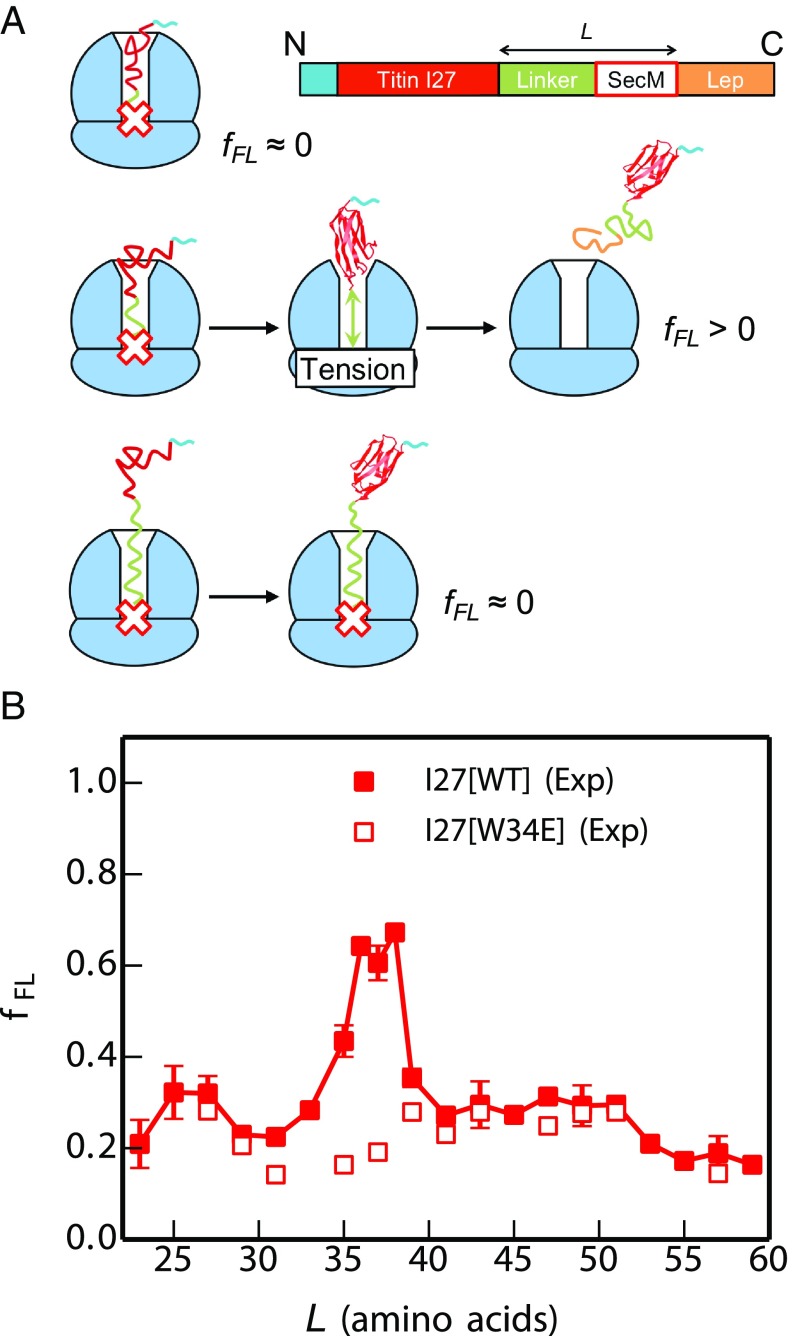
Cotranslational folding of the titin I27 domain by force-profile analysis. (*A*) The force-measurement assay. Modified with permission from ref. 2. I27, preceded by a His-tag, is placed *L* residues away from the last amino acid of the SecM AP, which in turn is followed by a 23-residue C-terminal tail derived from *E. coli* LepB. Constructs are translated for 15 min in the PURE in vitro translation system, and the relative amounts of arrested and full-length peptide chains produced are determined by SDS/PAGE. The fraction full-length protein, *f*_*FL*_, reflects the force exerted on the AP by the folding of I27 at linker length *L*. At short linker lengths (*Top*) there is not enough room in the exit tunnel for I27 to fold, little force is exerted on the AP, and the ribosome stalls efficiently on the AP (*f*_*FL*_ ≈ 0). At intermediate linker lengths (*Middle*) there is enough room for I27 to fold but only if the linker segment is stretched, force is exerted on the AP, and stalling is reduced (*f*_*FL*_ > 0). At long linker lengths (*Bottom*) I27 has already folded when the ribosome reaches the last codon in the AP, and again little force is exerted on the AP (*f*_*FL*_ ≈ 0). (*B*) Force profiles for the I27 domain (solid squares) and the nonfolding (nf) mutant I27[W34E] (open squares). The standard error of *f*_*FL*_ is calculated for values of *L* where three or more experiments were performed.

To gain insight into when I27 can commence folding on the ribosome, we employed an AP force-measurement assay (28) carried out using the PURE in vitro translation system, as described in refs. 1–3. In these experiments, the *Escherichia coli* SecM AP is used to stall the nascent protein chain temporarily during translation. The yield of full-length protein which escapes stalling in a defined time interval (*f*_*FL*_), determined from SDS/PAGE gels, provides a proxy for the pulling force exerted on the nascent chain by the protein as it folds (1–3) (Fig. 1*A*). By measuring *f*_*FL*_ for a set of constructs where the length *L* of the linker between the target protein and the SecM AP is systematically varied, a force profile can be recorded that reflects the points during translation where the folding process starts and ends. Previous work has shown that the location of the main peak in a force profile correlates with the acquisition of protease resistance in an on-ribosome pulse-proteolysis assay (17, 29) and that the amplitude of the main force peak correlates with the thermodynamic stability of the protein (29, 30), indicating that the main peak represents a bona fide folding event rather than, for example, the formation of a molten globule. The sharp onset of the main force peak observed for most proteins analyzed thus far (29) is also as expected for a cooperative folding event.

The force profile for wild-type I27 (Fig. 1*B*) has a distinct peak at *L* = 35–38 residues (see *Materials and Methods* for sequences of the constructs). This peak is absent from the force profile for the mutant I27[W34E], a nonfolding variant of I27, demonstrating that the peak is due to a folding event and not, for example, to nonspecific interactions of the unfolded nascent chain with the ribosome. The nonzero *f*_*FL*_ for the nonfolding mutant is attributed to the spontaneous rate of escape from arrest in the absence of acceleration by forces associated with folding. Since it takes ∼35 residues in an extended conformation to span the ∼100-Å long exit tunnel (31), the critical length *L* ≈ 35 residues suggests that I27 starts folding while in the mouth of the exit tunnel.

### Cryo-EM Shows That I27 Folds in the Mouth of the Exit Tunnel.

**Fig. 2.**
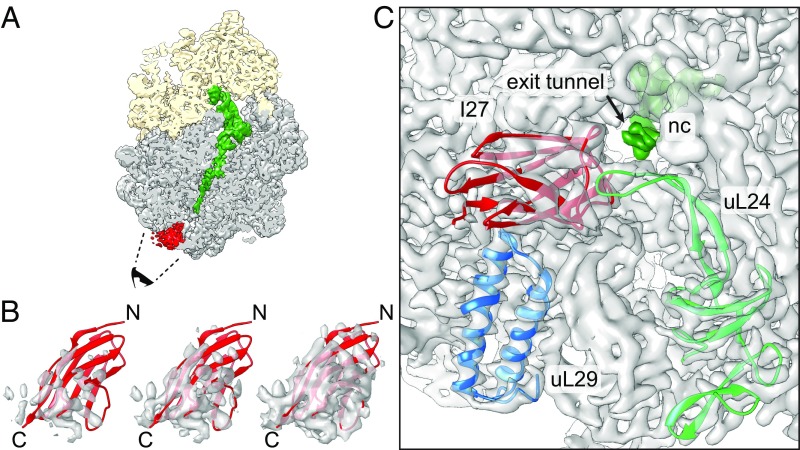
Cryo-EM structure of I27[*L* = 35] RNCs. (*A*) Cryo-EM reconstruction of the I27–TnaC[*L* = 35] RNC. The ribosomal small subunit is shown in yellow, the large subunit in gray, the peptidyl-tRNA with the nascent chain in green, and an additional density corresponding to I27 at the ribosome tunnel exit in red. The black cartoon eye and dashed lines indicate the angle of view in *C*. The density contour level for feature visualization is at 1.7 times rmsd. (*B*) Rigid-body fit of the I27 domain (PDB ID code 1TIT) to the cryo-EM density map displaying from high (*Left*) to low (*Right*) contour levels at 2.6, 2.0, and 1.4 rmsd, respectively. N and C represent the N and C termini of the I27 domain, respectively. (*C*) View looking into the exit tunnel (arrow) with density for the nascent chain (nc) in dark green. Ribosomal proteins uL29 (blue; PDB ID code 4UY8), uL24 (light green; the β hairpin close to I27 domain was remodeled based on PDB ID code 5NWY) and the fitted I27 domain (red) are shown in cartoon mode; 23S RNA and proteins not contacting I27 are shown as density only. The density contour level is at 5 rmsd excluding tRNA, nascent chain, and I27 domain, which are displayed at 1.7 rmsd.

To confirm that the peak in the force profile corresponds to the formation of a folded I27 domain, we replaced the SecM AP with the stronger TnaC AP (32–34) and purified stalled ribosome-nascent chain complexes (RNCs) carrying an N-terminally His-tagged I27[*L* = 35] construct (*Materials and Methods*). The construct was expressed in *E. coli*, RNCs were purified using the N-terminal His-tag, and an RNC structure with an average resolution of 3.2 Å (*SI Appendix*, Fig. S1) was obtained by cryo-EM. In addition to the density corresponding to the TnaC AP, a well-defined globular density (∼4.5- to 9-Å resolution) was visible protruding from the exit tunnel (Fig. 2*A*). Given the flat ellipsoidal shapes of the protruding density and of the I27 structure, there is only one way to fit the NMR structure of I27 [Protein Data Bank (PDB) ID code 1TIT (35)] that gives a good Fourier-shell correlation between the isolated I27 density and the map generated from the I27 PDB model (*SI Appendix*, Fig. S2). In the fitted model, the C-terminal end of I27 extends into the exit tunnel and a β-hairpin loop on ribosomal protein uL24 is lodged in a cavity in I27 (Fig. 2*B* and Movie S1). The I27 domain further packs against ribosomal protein uL29 and ribosomal 23S RNA (Fig. 2*C*), as if it is being pulled tight against the ribosome by the nascent chain. We conclude that the peak at *L* = 35–38 residues in the force profile indeed represents the cotranslational folding of the I27 domain at the tunnel exit.

### Coarse-Grained Molecular Dynamics Simulations Recapitulate I27 Folding on the Ribosome.

**Fig. 3.**
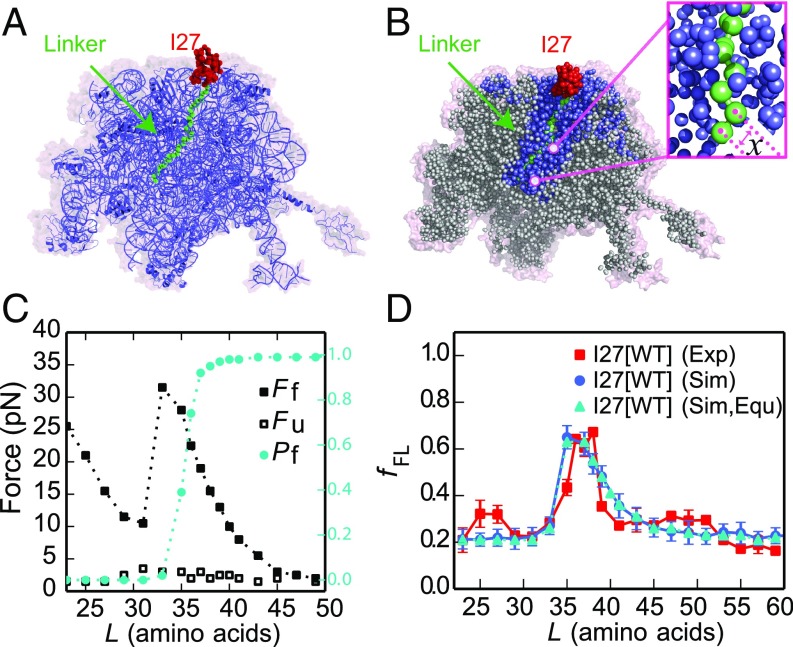
MD simulations of cotranslational folding of I27. (*A*) The 50S subunit of the *E. coli* ribosome (PDB ID code 3OFR) with I27[*L* = 35] attached via an unstructured linker. (*B*) Coarse-grained model for I27 (red) and linker (green), with surrounding ribosomal pseudoatoms in blue. Pseudoatoms colored gray are not used in the simulations. The instantaneous force exerted on the AP is calculated from the variation in the distance *x* between the C-terminal Pro pseudoatom and the next pseudoatom in the linker (*Inset*). (*C*) Average forces exerted on the AP by the unfolded state ($F_{u}$, empty symbols) and folded state ($F_{f}$, filled symbols) of I27 at different linker lengths *L*. The average fraction folded I27 for different *L*$,P_{f}$, is shown in cyan on the right axis. Free energy profiles at each linker length are shown in *SI Appendix*, Fig. S11. (*D*) Experimental (red square) force profiles for cotranslational folding of I27. Force profiles calculated from simulations using the full kinetic scheme or preequilibrium model are shown by blue circles and cyan triangles, respectively. The rmsd of the $f_{FL}$ between experiment and simulation is 0.08.

The yield of folded protein in AP experiments has been used as a proxy for the pulling forces that are exerted on the nascent chain at different points during translation in all studies to date (1, 2, 29). Here, to further elucidate the molecular origins of these forces and provide a quantitative interpretation of the observed folding yield of I27, we have calculated force profiles based on coarse-grained molecular dynamics (MD) simulations (*Materials and Methods*). Briefly, in the MD model, the 50S subunit of the *E. coli* ribosome (36) (PDB ID code 3OFR) and the nascent chain are explicitly represented using one bead at the position of the Cα atom per amino acid and three beads (for P, C4′, and N3) per RNA base (Fig. 3*A*). The interactions within the protein were given by a standard structure-based model (37–40), which allowed it to fold and unfold. Interactions between the protein and ribosome beads were initially purely repulsive (41) and the ribosome beads were fixed in space, as in previous simulation studies (18). I27 was covalently attached to unstructured linkers having the same sequences as those used in the force-profile experiments (Fig. 3*B*) and the C terminus of the linker was tethered to the last P atom in the A-site tRNA (42) with a harmonic potential, allowing the force exerted by the folding protein to be directly measured. The potential chosen was stiff enough that displacements caused by typical pulling forces were smaller than 1 Å. For each linker length *L*, we used umbrella sampling to determine the average force exerted on the AP by the protein in the folded and unfolded states while arrested, as well as the populations of those two states (Fig. 3*C*). We also estimated the folding and unfolding rates directly from folding/unfolding simulations.

Given the experimentally determined force dependence of the escape rate *k*(*F*) (25), here approximated by a Bell-like model (43), we can calculate the expected escape rate while the protein is in the unfolded or folded state. Taken together with the linker length-dependent folding and unfolding rates, this allows the fraction full-length protein obtained with a given linker length and incubation time to be determined from a kinetic model, as described in *Materials and Methods* (see also *SI Appendix*, Fig. S3). The calculated *f*_*FL*_ profile for I27 is shown in Fig. 3*D* using both full solution of the kinetic model, as well as for an approximation in which the folding and unfolding rates are assumed to be faster than the escape rate (“preequilibrium”). Both results are very consistent with each other, as well as with the experimental profile. The agreement between the two solutions of the kinetic model suggests that the preequilibrium assumption is reasonable. The peak in the folding yield arises as a consequence of two opposing effects, the force exerted by the folded protein and population of the folded state, which respectively decrease and increase as the linker length increases. In the simulations with the I27[*L* = 35] construct, the folded I27 domain is seen to occupy positions that largely overlap with the cryo-EM structure (Movie S2). Overall, these results suggest that the MD model provides a good representation of the folding behavior of the I27 domain in the ribosome exit tunnel. To show that the simulation model is not specific to I27, we have also applied it to another two proteins with different topologies for which experimental force profiles have been recorded, Spectrin R16 (all-α fold) and S6 (α/β fold) (2, 29). In these cases, we also obtain force profiles similar to experiment (*SI Appendix*, Figs. S4 and S5).

### Force Profiles of I27 Variants Probe the Folding Pathway.

**Fig. 4.**
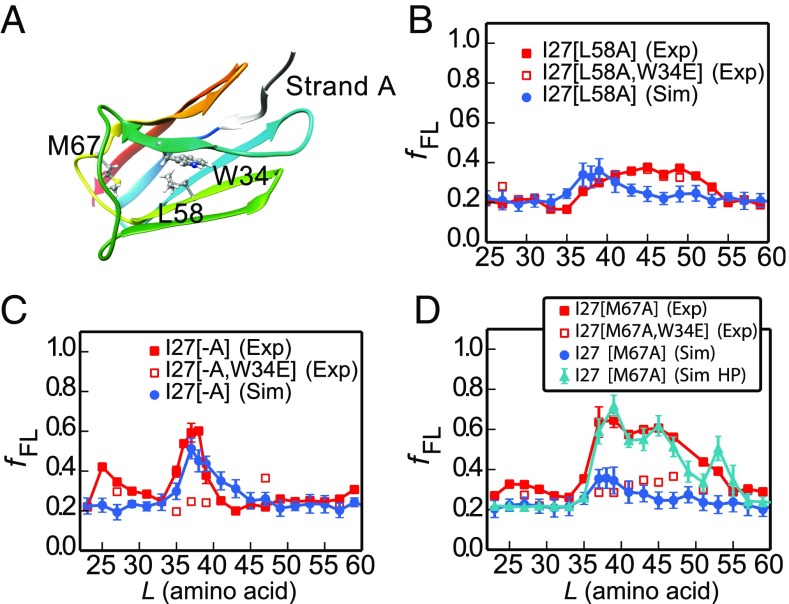
Simulations capture the experimental force profiles for mutant I27 domains. (*A*) Mutated residues in I27 (sticks). (*B*–*D*) Experimental (red) force profiles and calculated ones from the full kinetic scheme (blue) for (*B*) I27[L58A], (*C*) A-strand deletion mutant I27[−A], and (*D*) I27[M67A]. I27[M67A] (Sim HP) represents a simulation in which hydrophobic interactions between I27[M67A] and ribosome proteins uL23/uL29 are included. Experimental force profiles for nonfolding mutants that contain an additional W34E mutation are shown as open red squares. The rmsd of the $f_{FL}$ between experiment and simulation for I27[L58A] and I27[−A] are 0.07 and 0.08, respectively. For I27[M67A], the $f_{FL}$ rmsd is 0.07 between experiment and simulation (Sim HP).

To test whether the cotranslational folding pathway is the same as that observed for the isolated I27 domain in vitro, we investigated three destabilized variants of I27, both by simulation and experiment. One mutation in the core, Leu-58 to Ala (L58A), located in β-strand E (Fig. 4*A*), destabilizes the protein by 3.2 kcal⋅mol^−1^ and removes interactions that form early during folding of the isolated domain, playing a key role in formation of the folding nucleus (ϕ-value = 0.8) (26). Two further mutations, M67A and deletion of the N-terminal A-strand, remove interactions that form late in the folding of I27 [i.e., both mutants have low ϕ-values (26, 27)]. The A-strand is the first part of I27 to emerge from the ribosome, while M67 is located in a part of I27 that is shown by cryo-EM to be located in very close proximity to a β hairpin loop of ribosomal protein uL24 in I27-TnaC[*L* = 35] RNCs (*SI Appendix*, Fig. S6*A*). The interaction with the I27 domain shifts the tip of this uL24 hairpin by about 6 Å compared with its location in other RNC structures (*SI Appendix*, Fig. S6*B*).

The simulated force profile for the L58A variant predicts a much lower force peak than for wild-type I27; likewise, the experimental force peak is lower and broader than for wild type, extending from *L* = 37 to 53 residues (Fig. 4*B*). The *f*_FL_ values are very similar to those obtained for I27[L58A,W34E], a nonfolding variant of I27[L58A]. Therefore, the weak forces seen at *L* ≈ 40–50 residues are not due to a folding event, indicating that I27[L58A] does not exert an appreciable force due to folding near the ribosome.

The A-strand comprises the first seven residues of I27 and removal of this strand, I27[−A], results in a destabilization of 2.78 kcal⋅mol^−1^; however, both the simulated and experimental force profiles for I27[−A] are very similar to those for wild-type I27 (Fig. 4*C*). Residue M67 is located in the EF loop, and mutation to alanine results in a destabilization of 2.75 kcal⋅mol^−1^; for this variant, folding commences at *L* ≈ 35 residues as for wild-type I27, but the peak is much broader (Fig. 4*D*). Nonfolding control experiments for variants I27[−A,W34E] and I27[M67A,W34E] (Fig. 4 *C* and *D*) show that the peaks in the force profiles for these variants are due to a folding event. These results show that deletion of the A-strand and destabilization of the EF loop do not affect the onset of cotranslational folding of I27, but that the M67A mutation increases the width of the folding transition. The simulation model used for the other mutants does not predict such a broad peak, suggesting that it may be necessary to include additional factors to reproduce the data for M67A. One possibility which may explain the result would be favorable interactions of the folded M67A mutant with the ribosome surface. The ribosomal surface proteins uL23 and uL29 have been suggested to form a potential interaction site for nascent proteins such as trigger factor (44), signal recognition particle (45), and SecYE (46). Here we have explored the hypothesis that the broad force peak of mutant M67A might be due to interactions between an exposed hydrophobic cavity on I27[M67A] resulting from the mutation and hydrophobic surface residues of ribosomal proteins uL23 and uL29. By introducing such interactions into the model, we are able to obtain a broad peak in the force profile very similar to that seen in experiment (Fig. 4*D*). We have characterized the specific hydrophobic residues on the ribosome surface forming contacts with I27[M67A] from equilibrium simulations at a linker length of *L* = 45 (*SI Appendix*, Fig. S7), finding that residues 50, 51, and 93 from uL23 and residue 22 from uL29 have the most frequent interactions with I27[M67A] (residue numbering based on PDB ID code 3OFR).

### The Folding Pathway Is only Subtly Affected by the Presence of the Ribosome.

**Fig. 5.**
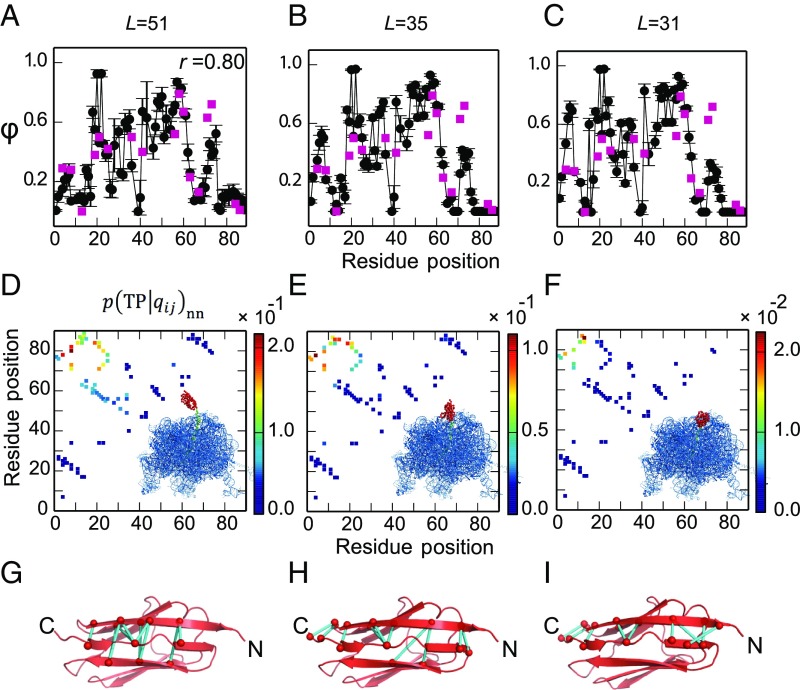
Simulated folding pathways for ribosome-tethered I27 (*Left*, *L* = 51; *Middle*, *L* = 35; *Right*, *L* = 31). (*A*–*C*) Simulated ϕ-values for I27 (black). ϕ-Values determined by in vitro folding of purified I27 are shown as red squares. At *L* = 51 the simulated ϕ-values match well with experiment (Spearman correlation coefficient *r* = 0.80). At *L* = 35 and *L* = 31 the simulated ϕ-values are higher at the N terminus and lower at the C terminus than the experimental values, reflecting a change in importance of these regions when I27 folds in the confines of the ribosome. (*D*–*F)* Relative probability that if a particular contact is formed then the protein is on a folding trajectory, $p{\left(TP|q_{ij}\right)}_{\text{nn}}$. When the protein is constrained the limiting factor is formation of a few key contacts. A cartoon of the ribosome with I27 in red is shown in each panel. (*G*–*I*) The top 10 most important contacts are colored in cyan on the native structure.

To compare the folding pathways when the protein is folding near the tunnel exit or outside the ribosome, we estimated ϕ-values based on the transition paths (TPs) of I27 folding on the ribosome from our coarse-grained simulations, using a method introduced previously (47). The TPs are those regions of the trajectory where the protein crosses the folding barrier, here defined as crossing between *Q* = 0.3 and *Q* = 0.7. For each linker length, 30 TPs were collected from MD simulations. To reduce the uncertainty in the experimental reference data, we only compared with experimental ϕ-values if the change in folding stability between the mutant and the wild type is sufficiently large (|ΔΔG| > 7 kJ/mol) (48). As seen in Fig. 5*A*, when the linker is long (*L* = 51 residues) and I27 is allowed to fold outside the ribosome, the calculated ϕ-values are consistent (Spearman correlation *r* = 0.80) with the experimental values obtained for the folding of isolated I27 in vitro (26). For shorter linker lengths (*L* = 31 and 35 residues), calculated ϕ-values remain largely unchanged except for a slight increase near the N terminus (around residues 3–6) and a slight decrease near the C terminus (around residues 72–74) (Fig. 5 *B* and *C*).

In most of our simulations we have treated interactions between I27 and the ribosome as repulsive. However, for the mutant I27[M67A] we found that incorporating hydrophobic interactions between I27 and the ribosomal proteins uL23 and uL29 better reproduced the experimental fraction full length (Fig. 4*D*). This naturally leads one to ask whether such interactions may affect the folding pathway. To address this, we investigated the folding mechanism of I27[M67A] at *L* = 45 (at the center of the broad *f*_FL_ peak) as well as two other linker lengths, *L* = 35 and *L* = 51 (on the edges of the *f*_FL_ peak). The results (*SI Appendix*, Fig. S8) indicate that the folding pathway of I27[M67A] is only subtly affected by the hydrophobic interaction with the ribosome, and the folding pathway of I27[M67A] is conserved at different linker lengths. This is most likely because the folding mechanism of I27 is fairly homogeneous with respect to the involvement of residue 67, as indicated by the relatively small variations in calculated ϕ-values between different folding transition events (standard deviation of ϕ-value of residue 67 = 0.1), and hence is less susceptible to perturbation.

To obtain a more detailed picture regarding the relative importance of different native contacts in the folding mechanism, we computed the conditional probability of being on a TP, given the formation of a contact $q_{ij}$ between residues *i* and *j*, $p{\left(TP|q_{ij}\right)}_{\text{nn}}$ (49). This quantity indicates which native contacts are most important for determining a successful folding event. $p{\left(TP|q_{ij}\right)}_{\text{nn}}$ is closely related to the frequency of the contact *q*_ij_ on TPs $p\left(q_{ij}|TP\right)$ but is effectively normalized by the probability that the contact is formed in nonnative states $p{\left(q_{ij}\right)}_{\text{nn}}$ and can be expressed as

$$p{\left(\text{TP|}q_{ij}\right)}_{\text{nn}}=\frac{p\left(q_{ij}\text{|TP}\right)p{\left(\text{TP}\right)}_{nn}}{p{\left(q_{ij}\right)}_{\text{nn}}},$$ [1]

where $p{\left(\text{TP}\right)}_{nn}$ is the fraction of nonnative states which are on TPs at equilibrium. The subscript *nn* means that only the nonnative segments of a trajectory are included (i.e., unfolded states and TPs); the native, folded state is not included in the calculation since native contacts are always formed in this state. The simulations suggest that formation of native contacts between the N and C termini is somewhat more important when folding takes place in the mouth of the exit tunnel (*L* = 31 residues) than far outside the ribosome (*L* = 51 residues) (Fig. 5 *D*–*F*, upper left-hand corners). This is likely due to the greater difficulty of forming these contacts (examples are shown in Fig. 5 *G*–*I*) under ribosomal confinement; therefore, forming them becomes more critical in enabling the protein to fold.

## Discussion

Using a combination of MD simulation, force-profile measurements, and cryo-EM, we have investigated the cotranslational folding pathway of the 89-residue titin I27 domain. I27 has been extensively characterized in previous in vitro folding studies (26, 27, 50–61). Results from all three techniques show that wild-type I27 folds in the mouth of the ribosome exit tunnel; in the cryo-EM structure of I27-TnaC[*L* = 35] RNCs, I27 packs against ribosomal proteins uL24, uL29, and ribosomal 23S RNA. This is in apparent contrast to a previous NMR study on another Ig-like protein, in which the domain was shown to acquire its native fold (as reflected in the NMR spectrum) only when fully outside the ribosome tunnel, at *L* = 42–47 residues linker length (20).

To determine the molecular origin of the measured force profile, we performed MD simulations of I27 folding on the ribosome, varying the length of the linker sequence between the AP and the I27 domain. We calculated the pulling force directly from the simulations and translated this into yield of folded protein using a kinetic model parameterized based on known release kinetics of the SecM AP. This enabled us to recapitulate the experimental AP force measurement profile and therefore relate *f*_*FL*_ directly to the force exerted on the AP. Our simulations demonstrate the direct effect that the restoring force of the nascent chain can have on determining when the protein folds on the ribosome. We show that *f*_*FL*_ depends upon a combination of the force exerted by the folded protein and the fraction of folded protein at the given linker length *L*.

To relate how destabilization of regions that fold early and late in the isolated domain affects folding on the ribosome, we used simulations to predict the onset of folding in three mutant variants of I27. A previous ϕ-value analysis of I27 (26) showed that early packing of the structurally central β-strands drives the folding of this domain, while peripheral strands and loop regions pack later in the folding process. Mutations in the folding core (such as L58A) slow folding, whereas mutations in the periphery have no effect on folding rates (26). L58 is a key residue in the critical folding nucleus and almost fully packed in the transition state, in isolated domain studies. The simulated and experimental force profiles of I27[L58A] show that this variant does not fold in or near the exit tunnel; hence, destabilization of the central folding core prevents folding close to the ribosome. Since isolated I27[L58A] is fully folded, it is likely that this variant can only fold cotranslationally at longer linker lengths, when it is no longer in close proximity to the ribosome and exerts little force on the nascent chain.

Our experiments show that I27 variants destabilized in regions of the protein that are unstructured, or only partially structured, in the transition state, are still able to commence folding close to the ribosome. The force profiles reveal that the onset of folding of mutants with the A-strand deleted, or with the Met-67 to Ala mutation in the EF loop, is the same as for wild type, although these have a destabilization similar to that of L58A (Fig. 4). The broader peak observed experimentally for M67A is harder to interpret. A plausible explanation is that the mutation introduces nonspecific interactions of the folded domain with the ribosome surface. We have shown that, by incorporating interactions between exposed hydrophobic residues of ribosomal proteins and the mutated site on I27, we could reproduce the experimental fraction full length for this mutant. There is direct evidence for favorable interactions between I27 and the ribosome, albeit at a shorter linker length, from the wild-type cryo-EM structure, in which ribosomal protein uL24 has shifted to form contacts with I27 (close to residue 67). Specifically, residues from V49 to N53 at the tip of uL24 loop are in contact with I27 as shown in *SI Appendix*, Fig. S6. A recent study has also shown that I27 can fold closer to the ribosome tunnel mouth after deletion of residues 43–57 from this loop of uL24 (62).

Our simulations reproduce the onset of folding in the three mutant variants of I27 (Fig. 4) and so give us the confidence to investigate how confinement within the ribosome affects the folding pathway of I27. We used simulations to investigate the folding of I27 arrested on the ribosome at various linker lengths, using a Bayesian method for testing the importance of specific contacts on the folding pathway, as well as by computing ϕ-values (Fig. 5). Overall, we find that the mechanism and pathway of folding are robust toward variation in linker length and relatively insensitive to the presence of the ribosome; small but significant changes are observed only for contacts near the N and C termini. These shifts are consistent with the greater importance of forming N-terminal contacts when the C terminus is sequestered within the exit tunnel, possibly to compensate for loss of contacts at the C terminus.

In our kinetic modeling we found that we obtained similar results with or without the assumption that folding and unfolding are fast relative to the escape rate, suggesting that this preequilibrium assumption is justified, at least for this protein. The reason for its validity in the case of I27 can be seen by comparing the folding and unfolding rates with the force-dependent escape rate of ∼2.4 × 10^−3^ s^−1^ obtained at the highest forces of ∼20 pN (cf. Fig. 3*C*). Folding and unfolding rates at different linker lengths can be obtained by combining the linker length dependence of the rates from simulation with the known folding/unfolding rates for isolated I27 from experiment (*SI Appendix*, Fig. S9). The presence of the ribosome increases the unfolding rate at shorter linker lengths so that it is faster than the maximum escape rate, while not slowing the folding rate sufficiently for it to drop below the escape rate. Note that the unfolding rate does drop below the maximum escape rate at larger linker lengths, but by that point the folded population is already almost 100%, so the preequilibrium assumption still gives accurate results. Although this assumption appears to be justified in the case of I27, it is probably not true in general, and it will be interesting to investigate for slower-folding proteins in the future.

The AP experiments, in which a protein exerts a force due to folding, in some ways resemble atomic force microscopy or optical tweezer experiments, in which an external force is applied to the protein termini. It is important to note, however, that the nature and effect of the forces exerted on the folding protein by tethering to the ribosome are very different from the case for pulling on both termini by an external force. For example, forces of the magnitude seen in this work (up to ∼20 pN) tend to have very little effect on the unfolding rate when applied to the termini of I27, due to the similarity in extension of the folded and transition states (63); by contrast, folding rates are dramatically slowed, even by very small forces, due to the large difference in extension between unfolded and transition states (57). The forces arising from tethering to the ribosome are due to the folding of the protein itself rather than an external device. They arise from the constriction of available configuration space, particularly for folded and partially folded states, as well as from any additional attractive or repulsive interactions between the protein and the ribosome. Our simulations suggest that for I27 reducing the linker length speeds up unfolding and slows folding rates by similar factors. Thus, it is clear that comparisons to the effects of forces exerted by atomic force microscopy and optical tweezer experiments need to be performed with care.

We have previously shown that α-helical proteins can fold cotranslationally (2), which is perhaps unsurprising since helical structures are dominated by short-range interactions and helices can form within the ribosome tunnel itself (64, 65). Here, our equilibrium AP assay and structural studies reveal that an all-β protein, titin I27, is able to fold within the mouth of the ribosome exit tunnel, despite its folding being dominated by long-range interactions. Molecular simulations, accounting for the effect of the entropic restoring force on protein stability, reproduce the yield of protein from experiments remarkably well. These simulations reveal that I27 folds on the ribosome by the same pathway as when the protein folds away from the confines of the ribosome. We note that a similar conclusion has been reached by Guinn et al. (66) for another small protein, src SH3, using a completely different experimental approach which combines optical tweezer experiments and chemical denaturant to characterize the folding pathway of src SH3. Thus, the evidence so far suggests that single-domain proteins, both α-helical and β-sheet, can fold close to the ribosome. However, while all-β proteins appear to fold by a similar pathway with or without the ribosome present, there is evidence for α-helical proteins forming partially structured cotranslational intermediates (11, 67) or folding by different pathways on the ribosome (2). This mechanistic difference may relate partly to the small contact order of helical proteins, allowing partially folded states to be more stable than for all-β proteins. The situation for multidomain proteins is likely to be still more complicated, as some studies have already indicated (11, 23, 68, 69). However, it seems likely that folding of individual domains close to the ribosome should help to avoid the formation of misfolded species involving interdomain interactions. In particular, we have previously shown the occurrence of both domain-swapped and other misfolded states in multidomain constructs of I27, after refolding from chemical denaturants (70, 71). It will be interesting to investigate the role of cotranslational folding in preventing the formation of such aberrant folds.

## Materials and Methods

### Enzymes and Chemicals.

All enzymes were obtained from Thermo Scientific. Oligonucleotides were purchased from Life Technologies. In-Fusion Cloning kits were obtained from Clontech and DNA purification kits were purchased from Qiagen. PUREfrex cell-free translation system was obtained from Eurogentec. [^35^S]methionine was purchased from PerkinElmer. Instant Blue protein stain was purchased from Expedeon.

### DNA Manipulation.

Titin I27 constructs for in vitro translation were generated in pRSET A plasmid (Invitrogen) (previously modified to remove the sequence including the entire T7 gene 10 leader and EK recognition site up to, but not including, the BamH I site and replaced with a sequence encoding residues L, V, P, R, G, and S) carrying the *E. coli* SecM AP (FSTPVWISQAQGIRAGP) and a truncated *E. coli lepB* gene, under the control of a T7 promoter. Increasing linker lengths were generated in pRSET A by PCR; linear pRSET A constructs (containing the SecM AP and truncated lepB, but lacking I27) were generated by PCR using primers which extended the linker from 23 aa to 63 aa (in steps of 2 aa) from the direction of the C to the N terminus. I27 flanked by GSGS linkers was amplified by PCR with overhanging homology to the plasmid containing the desired linker length. Cloning was performed using the In-Fusion system (Takara Bio USA, Inc.), according to the manufacturer’s instructions. The final two C-terminal residues (EL) of the 89-aa Titin I27 construct are not structured in the PDB file 1TIT and are therefore included in the linker region. The amino acid sequence of the construct I27[*L* = 63] is as follows (I27 in bold and SecM AP underlined): MRGSHHHHHHGLVPRGSGS**LIEVEKPLYGVEVFVGETAHFEIELSEPDVHGQWKLKGQPLAASPDCEIIEDGKKHILILHNCQLGMTGEVSFQAANTKSAANLKVK**ELSGSGKFAYGIKDPIYQKTLVPGQQNATWIVPPGQYFMMGDWMSSFSTPVWISQAQGIRAGPGSSDKQEGEWPTGLRLSRIGGIH**.

The mutants I27[–A] (lacking β-strand A), I27[L58A], and I27[M67A] were generated for each linker length by site-directed mutagenesis. For the wild-type I27 and I27[–A] constructs with *L =* 27, 35, 37, 39, 47, and 57 residues, site-directed mutagenesis was performed to generate constructs with the nonfunctional FSTPVWISQAQGIRAGA AP (mutated residue underlined) as full-length controls and constructs with the crucial Pro, at the end of the AP, substituted with a stop codon as arrest controls. Site-directed mutagenesis was performed to generate W34E variants as nonfolding (nf) controls at *L* = 27, 29, 31, 35, 37, 39, 41, 43, 47, 49, 51, and 57 for wild-type I27; *L* = 27, 35, 37, 39, 47, and 57 residues for I27[–A]; *L* = 27, 41, 45, 47, 49, and 53 residues for I27[L58A]; and *L* = 27, 29, 37, 39, 41, 43, 45, 47, and 51 residues for I27[M67A]. All constructs were verified by DNA sequencing.

### In Vitro Transcription and Translation.

Transcription and translation were performed using the commercially available PUREfrex in vitro system (GeneFrontier Corporation), according to the manufacturer’s protocol, using 250 μg plasmid DNA as template. Synthesis of [^35^S]Met-labeled polypeptides was performed at 37 °C, 500 rpm for exactly 15 min. The reaction was quenched by the addition of an equal volume of 10% ice-cold trichloroacetic acid. The samples were incubated on ice for 30 min and centrifuged for 5 min at 20,800 × *g* and 4 °C. Pellets were dissolved in sample buffer and treated with RNase A (400 μg⋅mL^−1^) for 15 min at 37 °C before the samples were resolved by SDS/PAGE and imaged on a Typhoon Trio or Typhoon 9000 phosphorimager (GE Healthcare). Bands were quantified using ImageJ to obtain an intensity cross-section, (https://imagej.nih.gov/ij/), which was subsequently fit to a Gaussian distribution using in-house software (Kaleidagraph; Synergy Software). The fraction full-length protein, *f*_*FL*_, was calculated as *f*_*FL*_ = *I*_*FL*_/(*I*_*FL*_ + *I*_*A*_), where *I*_*FL*_ and *I*_*A*_ are the intensities of the bands representing the full-length and arrested forms of the protein. For wild-type I27 and six nf control samples (*L* = 27, 35, 37, 39, 47, and 57 residues), in vitro transcription and translation were also performed at 37 °C, 500 rpm for exactly 30 min. The resultant force profile was slightly higher than that obtained at 15 min but has essentially the same shape (*SI Appendix*, Fig. S3).

The reproducibility of force profile data has been discussed previously (2). For wild-type I27, data points *L* = 61 and 63 residues are a single experiment; *L* = 33, 36, 38, 45, 53, 55, and 59 residues are an average of two experiments; all other values of *L* are an average of at least three experiments. For I27[–A] strand, *L* = 23, 25, 33, 41, 43, 51, 53, and 55 residues are a single experiment; all other values of *L* are an average of two experiments, except *L* = 35, 37, and 39 residues, which are an average of at least three experiments. For I27[L58A], all data points are a single experiment except *L* = 27, 37, 41, 45, 47, 49, and 53 residues, which are an average of two experiments. For I27[M67A], *L* = 23, 25, and 51–63 residues are a single experiment; *L* = 29–35 residues are an average of two experiments; *L* = 27, 37–47, and 51 residues are an average of at least three experiments. For wild-type I27 samples incubated for 30 min, all data points are a single experiment except *L* = 27, 35, 37, 39, 47, and 57 residues, which are an average of two experiments. For nonfolding controls, all data points are a single experiment except for wild-type I27 *L* = 29, 31, 39, 43, and 47 residues, which are an average of two experiments.

### Cloning and Purification of RNCs.

The I27 construct at *L* = 35, which is at the peak of *f*_*FL*_ (Fig. 1*B*), was studied by cryo-EM. The SecM AP in these constructs was substituted with the TnaC AP (34) for more stable arrest, and the constructs were engineered to maintain a linker length of 35 aa residues. An N-terminal 8X His tag was introduced to enable purification. The amino acid sequence of the construct used was (I27 in bold and TnaC AP underlined) as follows: MDMGHHHHHHHHDYDIPTTLEVLFQGPGTLIEVEKPLYGVEVFVGETAHFEIELSEPDVHGQWKLKGQPLAASPDCEIIEDGKKHILILHNCQLGMTGEVSFQAANTKSAANLKVKELSGSGSGSGGPNILHISVTSKWFNIDNKIVDHRP**.

The construct was engineered into a pBAD expression vector, under the control of an arabinose-inducible promoter. The translation-initiation region was optimized as described in ref. 72. The plasmid was transformed into the *E. coli* KC6 Δ*smpB* Δ*ssrA* strain. Four colonies were picked and tested for expression of the RNCs at 37 °C in LB.

Large-scale purification of RNCs was carried out based on a protocol described in ref. 34. Briefly, a single colony of the KC6 cells found to express the RNCs was picked and cultured in LB at 37 °C to an A_600_ of 0.5. Expression was induced with 0.3% arabinose and was carried out for 1 h. Thereafter, the cells were chilled on ice, harvested by centrifugation, and resuspended in buffer A at pH 7.5 (50 mM Hepes-KOH, 250 mM KOAc, 2 mM tryptophan, 0.1% DDM, and 0.1% complete protease inhibitor). Cell lysis was carried out by passing the cell suspension thrice through the Emulsifex (Avestin) at 8,000 psi at 4 °C. The lysate was cleared of cell debris by centrifugation at 30,000 × *g* for 30 min in the JA25-50 rotor (Beckman Coulter). The supernatant obtained was loaded on a 750 mM sucrose cushion (in buffer A) and centrifuged at 45,000 × *g* for 24 h in a Ti70 rotor (Beckman Coulter) to obtain a crude ribosomal pellet, which was resuspended in 200 μL buffer A by shaking gently on ice.

RNCs from the crude suspension were purified via their His tags by affinity purification using Talon (Clontech) beads, which were preincubated with 10 μg/mL tRNA to reduce unspecific binding of ribosomes. The suspension was incubated with the beads for 1 h at 4 °C and subsequently washed with 20 column volumes of buffer B at pH 7.5 [50 mM Hepes-KOH, 10 mM Mg(OAc)_2_, 0.1% complete protease inhibitor, 250 mM sucrose, and 2 mM tryptophan]. RNCs were eluted by incubating the Talon beads with buffer C at pH 7.5 [50 mM Hepes, 150 mM KOAc, 10 mM Mg(OAc)_2_, 0.1% complete protease inhibitor, 150 mM imidazole, and 250 mM sucrose] for 15 min and subsequently collecting the flow-through. Elution was carried out thrice and the eluents were concentrated by centrifugation at 40,000 rpm for 2.5 h in a TLA 100.3 rotor (Beckman Coulter). The pellet obtained at the end of this step was gently suspended in a minimal volume of buffer D at pH 7 [20 mM Hepes-KOH, 50 mM KOAc, 5 mM Mg(OAc)_2_, 125 mM sucrose, 2 mM Trp, and 0.03% DDM].

### Cryo-EM Sample Preparation, Data Collection, Processing, and Accession Codes.

Approximately 4 A_260_/mL units of RNCs were loaded on Quantifoil R2/2 grids coated with carbon (3 nm thick) and vitrified using the Vitrobot Mark IV (FEI-Thermo) following the manufacturer’s instructions. Cryo-EM data were collected at the Cryo-EM National Facility at the Science for Life Laboratory in Stockholm, Sweden.

Data were acquired on a 300-keV Titan Krios microscope (FEI) equipped with a K2 camera and a direct electron detector (both from Gatan). The camera was calibrated to achieve a pixel size of 1.06 Å at the specimen level. Thirty frames were acquired with an electron dose 0.926 e^−^/Å^2^ per frame and a total dose of 27.767 e^−^/Å^2^ and defocus values between −1 to −3 μm. The first two frames were discarded and the rest were aligned using MotionCor2 (73). Raw images were cropped into squares by RELION 2.1 beta 1 (74). Power spectra, defocus values, and estimation of resolution were determined using the Gctf software (75) and all 2,613 micrographs were manually inspected in real space, in which 2,613 were retained; 468,015 particles were automatically picked by Gautomatch (https://www.mrc-lmb.cam.ac.uk/kzhang/) using the *E. coli* 70S ribosome as a template. Single particles were processed by RELION 2.1 beta 1 (74). After 80 rounds of 2D classification, 384,039 particles were subjected to 3D refinement using the *E. coli* 70S ribosome as reference structure, followed by 160 rounds of 3D classification without masking and 25 rounds of tRNA-focused sorting. One major class containing 301,510 particles (64% of the total) was further refined including using a 50S mask, resulting in a final reconstruction with an average resolution of 3.2 Å (0.143 Fourier shell correlation). The local resolution was calculated by ResMap (76). Finally, the final map was obtained by local B-factoring followed by low-pass filtering to 4.5 Å by RELION 2.1 beta 1 (74) to best demonstrate the I27 domain.

For interpretation of the cryo-EM density, the cryo-EM structure model (PDB ID code 4UY8) of *E. coli* TnaC-stalled ribosome was fitted into corresponding density using UCSF Chimera (77). The NMR model (PDB ID code 1TIT) of I27 domain was fitted into the extra density of TnaC-stalled ribosome using UCSF Chimera (77). Since the I27 domain represents a flat ellipsoid, we used all four major and minor axes covering all possible orientations of the model fitting within the density to validate the orientation of the fitted I27 model. Briefly, the model with four different orientations was converted into densities (8 Å) by UCSF Chimera, and the cross-correlation coefficients of each model map and the isolated I27 density were calculated by RELION 2.1 beta 1 (74). Finally, uL24 β hairpin was remodeled as the tip of the hairpin is shifted due to the existence of I27 domain.

Figures showing electron densities and atomic models were generated using UCSF Chimera (77). Electron densities are shown at multiple contour levels in Fig. 2 and *SI Appendix*, Fig. S1. The contour levels relative to the rmsd were calculated from the final map values. The final map contains the volume for the entire RNC including the I27 domain.

Coordinates for the cryo-EM map of the ribosome with the I27 domain density have been deposited at the Electron Microscopy Data Bank under entry number 0322. Coordinates of fitted *E. coli* TnaC-stalled ribosome (PDB ID code 4UY8; uL24 remodeled) and I27 domain (PDB ID code 1TIT) models for interpreting the cryo-EM map have been deposited at the PDB under ID code 6I0Y.

### Coarse-Grained Molecular Simulations.

The 50S subunit of the *E. coli* ribosome [PDB ID code 3OFR (36)] and the nascent chain are explicitly represented using one bead at the position of the α-carbon atom of each amino acid and three beads (for P, C4′, and N3) per RNA residue (Fig. 3*B*). The interactions within the protein were given by a standard structure-based model (38–40), which allowed it to fold and unfold. Interactions between the protein and ribosome beads were purely repulsive (41) and given by the same form of potential as for the structure-based model (38–40),

$$V_{ij}=\varepsilon_{ij}\left[\frac{A}{{r_{ij}}^{12}}-\frac{B}{{r_{ij}}^{10}}+\frac{C}{{r_{ij}}^{6}}\right],$$ [2]

where $r_{ij}$ is the distance between two beads i and j and $\varepsilon_{ij}$ (= 0.001 kJ/mol) sets the strength of the repulsive interactions. The amino acid, phosphate, sugar, and base are assigned radii $\sigma_{i}=$ 4.5, 3.2, 5.1, and 4.5 Å, respectively, and coefficients in Eq. **2** for interactions between protein and ribosome beads *i*, *j* are obtained from the mixing rules *A* = $\sqrt{{\sigma_{i}}^{12}{\sigma_{j}}^{12}}$, *B* = $\frac{2}{{\sigma_{i}}^{-10}+{\sigma_{j}}^{-10}}$, and *C* = $\sqrt{{\sigma_{i}}^{6}{\sigma_{j}}^{6}}$.

During the simulations, the positions of the ribosome atoms were fixed in space, as in previous studies (18). The linker between the AP and I27 was tethered by its C terminus to the last P atom of the A-site tRNA, but was otherwise free to fluctuate. The trajectory was propagated via Langevin dynamics, with a friction coefficient of 0.1 ps^−1^ and a time step of 10 fs, at 291 K in a version of the Gromacs 4.0.5 simulation code, modified to implement the potential given by Eq. **2** (78). All bonds (except the one used to measure force, below) were constrained to their equilibrium length using the LINCS algorithm (79). The attractive interactions between I27[M67A] and the hydrophobic residues (A, V, L, I, F, M, Y, and W) on the surface of uL23 and uL29 are modeled as (80)

$$V_{ij}=4\varepsilon \left[{\left(\frac{\sigma }{r_{ij}}\right)}^{12}-{\left(\frac{\sigma }{r_{ij}}\right)}^{6}\right],$$ [3]

where $r_{ij}$ is the distance between residues *i* and *j*, σ is the range of the interaction, and ε represent the strength of the interaction. σ and ε are fixed at 6 Å and 5 kJ/mol, respectively. Residues of I27[M67A] which are involved in the attractive interactions are defined as the ones whose heavy atoms are within 4.5 Å of any heavy atoms from residue 67 in the native state. To model the mutant I27[M67A], the strength of all native contacts (I27 wild type) to residue 67 is weakened by the same factor (40%), so that the loss of folding stability (2.3 kcal/mol) was comparable to experiment (2.75 ± 0.1 kcal/mol).

To calculate the pulling force exerted on the nascent chain by the folding of I27, the bond between the last and the second-to-last amino acid of the SecM AP was modeled by a harmonic potential as a function of the distance between these two atoms, x (Fig. 3*B*):

$$E=\frac{1}{2}k_{\text{s}}{\left(x-x_{0}\right)}^{2}\;,$$ [4]

where $x_{0}$ is a reference distance. Here $x_{0}$ is set to 3.8 Å, which is the approximate distance between adjacent Cα atoms in protein structures and $k_{\text{s}}$ is a spring constant, set to 3,000 kJ⋅mol^−1^⋅nm^−2^. The value of $k_{\text{s}}$ was chosen so that the average displacement $x-x_{0}$ remains below 1 Å for forces up to ∼500 pN, which is much larger than the forces actually exerted by the folding protein. The pulling force on the nascent chain was measured by the extension of this bond as $F=-k_{\text{s}}\left(x-x_{0}\right)$.

I27 was covalently attached to unstructured linkers having the same sequences as used in the force-profile experiments (Fig. 1*B*). Linker amino acids are repulsive to both the ribosome and I27 beads, with interaction energy as described in Eq. **2**.

### Kinetic Model for Fraction Full Length *f*_*FL*_(*t*).

There is a free energy barrier for escape of the AP sequence from its trapped state near the peptide transfer center. The rate for escape over this barrier, $k_{e}$, can be accelerated by a force pulling on the end of the nascent chain, as has been experimentally demonstrated using optical tweezers (25). Such a force can also be exerted by a folding protein as it leaves the ribosomal exit tunnel and will fluctuate, for example when the protein folds or unfolds. Here, we approximate the sensitivity of the escape rate to a force *F* using the phenomenological expression originally proposed by Bell (43):

$$k_{e}\left(F\right)=k_{0}e^{\beta F\Delta x^{‡}},$$ [5]

where $k_{0}$ is a zero-force rupture rate, $\Delta x^{‡}$ is the distance from the free energy minimum to the transition state, and $\beta =1/k_{\text{B}}T$, where $k_{\text{B}}$ is Boltzmann’s constant and T the absolute temperature. While there are functions to describe force-dependent rates with a stronger theoretical basis, we use the Bell equation due to its simplicity and because its parameters have previously been estimated from optical tweezer experiments for the SecM AP (25). In all cases, we set $k_{0}$ (Eq. **5**) to 3.4 × 10^−4^ s^−1^ and $\Delta x^{‡}$ to 3.2 Å, based on the values determined by Goldman et al. (25) (they estimated $k_{0}$ and $\Delta x^{‡}$ to be in the range of 0.5 × 10^−4^ to 20 × 10^−4^ s^−1^ and 1–8 Å, respectively). These are the only free parameters (albeit chosen within the experimental bounds) in our model, but the same ones are used to describe all variants of I27, as well as the proteins shown in *SI Appendix*, Figs. S4 and S5.

We model the escape of I27 from the ribosome using the kinetic scheme shown in *SI Appendix*, Fig. S10. In this scheme, the protein can fold and unfold while attached to the ribosome, with folding and unfolding rates that depend on the linker length *L* [$k_{\text{f}}\left(L\right)$ and $k_{\text{u}}\left(L\right)$, respectively]. Each of the unfolded or folded state attached to the ribosome can also escape arrest with force-dependent rates $k_{e}(F_{\text{u}}\left(L\right))$ and $k_{e}(F_{\text{f}}\left(L\right))$, respectively, where $F_{\text{u}}\left(L\right)$ is the mean force exerted by the unfolded protein at linker length *L* and $F_{\text{f}}\left(L\right)$ the corresponding force exerted by the folded state. Note that $F_{\text{u}}\left(L\right)$ can be nonzero (81). These average forces are determined from the harmonic linker to the PTC, as described in the previous section, and $k_{e}\left(F\right)$ is given by Eq. **5**.

Thus, the only remaining parameters in our scheme are the folding/unfolding rates. To estimate $k_{\text{f}}$ at different linker lengths, we first carried out unbiased MD simulations to estimate the mean first passage time for folding $t_{\text{F}}^{\text{mfpt}}$, from which the folding rate can be calculated as $k_{\text{f}}^{\text{wt}}=1/t_{\text{F}}^{\text{mfpt}}$. Similarly, the unfolding rate can be calculated from unfolding simulations as $k_{\text{u}}^{\text{wt}}=1/t_{\text{u}}^{\text{mfpt}}$. Since the rates in coarse-grained simulations are naturally much faster than in experiment, we globally scale the unfolding rates $k_{\text{u}}^{\text{wt}}$ (*L* = 21, 23 …61) at different linker lengths so that $k_{\text{u}}^{\text{wt}}$ at very long linker lengths (*L* = 61) is equal to the unfolding rate of isolated I27 (4.9 × 10^−4^ s^−1^). Similarly, $k_{\text{f}}^{\text{wt}}$ (*L* = 21, 23 …61) is scaled so that the $k_{\text{f}}^{\text{wt}}$ of I27 RNC[*L* = 61] is equal to the unfolding rate of isolated I27 (*SI Appendix*, Fig. S9). For consistency with our preequilibrium solution, we further scale $k_{\text{f}}^{\text{wt}}$ to match the stability of I27 RNC[*L* = 61] in our simulation model, yielding $k_{\text{u}}^{\text{wt}}$ and $k_{\text{f}}^{\text{wt}}$ at *L* = 61 of 4.9 × 10^−4^ s^−1^ and 0.14 s^−1^ (*SI Appendix*, Fig. S9), respectively. The same scaling method has been applied to the folding and unfolding rates of all mutants (I27[L58A], I27[M67A], and I27[−A]) so that the folding/unfolding rates of the mutant RNC at large linker lengths are consistent with the experimental values measured for the isolated mutant domains (26). We assume the dependence of mutant folding/unfolding rates on linker length is the same as for wild type, an assumption we have verified by direct simulations of a mutant (I27[L58A]) in which the contacts to the mutated residue were weakened (*SI Appendix*, Fig. S9, *Insets*).

### *f*_*FL*_(*t*) Calculated by Full Kinetic Scheme.

Using the kinetic model with folding/unfolding rates $k_{\text{f}}^{\text{wt}}$, $k_{\text{u}}^{\text{wt}}$ and force-dependent escape rates from ribosome $k_{e}(F_{\text{u}}\left(L\right))$ and $k_{e}(F_{\text{f}}\left(L\right))$ at each linker length obtained from above, the time-dependent survival probability *S*(*t*) is estimated by the kinetic Monte Carlo method [the Bortz–Kalos–Lebowitz algorithm (82)]. The system is initialized at the state when the unfolded nascent chain just emerges from the ribosome tunnel (UA; *SI Appendix*, Fig. S10) at time *t* = 0. At each Monte Carlo step, a uniform random number δ between 0 and 1 is chosen, and a transition from the current state *s* to state *j* will occur for the state *j* which satisfies $\sum_{i=1}^{j-1}k_{si}<\delta \sum_{i=1}^{N}k_{si}<\sum_{i=1}^{j}k_{si}$, where $k_{si}$ represents the transition rate from state *s* to state *i*. The time is updated by t=t+Δt, where $\Delta t=-\left(\delta '\right)/\sum_{i=1}^{N}k_{si}$. δ' is a new number randomly chosen between 0–1. *f*_*FL*_(*t*) is the combined population in states UR and FR at time *t*.

### *f*_*FL*_(*t*) Calculated by the Preequilibrium Model.

The solution to the kinetic model can be simplified if we further assume that the escape from the ribosome is slow relative to the folding and unfolding of the protein. In this situation, we can approximate *f*_*FL*_(*t*) in terms of the mean forces experienced when the protein is unfolded, *F*_u_, or folded, *F*_f_, and the unfolded and folded populations of *P*_u_ and *P*_f_, respectively (note that each of these quantitites is implicitly dependent on the linker length *L*):

$$f_{FL}\left(t\right)\approx 1-\exp\left[-t\left[P_{\text{u}}k_{e}\left(F_{\text{u}}\right)+P_{\text{f}}k_{e}\left(F_{\text{f}}\right)\right]\right].$$ [6]

The equilibrium properties of the system for each linker length were obtained from umbrella sampling using the fraction of native contacts *Q* as the reaction coordinate, allowing $P_{\text{u}}$, $P_{\text{f}}$, and *F*_u_, *F*_f_ to be determined (Fig. 3*C*). In the umbrella sampling simulation, a harmonic potential well $V_{\text{umbrella}}=k_{\text{umb}}{\left(\text{Q}-{\text{Q}}_{0}\right)}^{2}/2$ was used in each of the 16 windows which span evenly along Q from 0 to 1. $k_{\text{umb}}$ = 600 kcal/mol. Equilibrium prosperities are obtained by reweighting the ensemble with WHAM (83). The details of the definition of *Q* have been previously described (49); in short, *Q* is defined as

$$Q=\frac{1}{N}\sum_{\left(i,j\right)}\frac{1}{1+e^{\gamma \left(r_{ij}-\lambda r_{ij}^{0}\right)}},$$ [7]

where the sum runs over the N pairs of native contacts (i,j), $r_{ij}$ is the distance between i and j in configuration, $r_{ij}^{0}$ is the distance between i and j in the native state, and *λ* = 1.2, which accounts for fluctuations when the contact is formed. The coefficient γ=50 nm^−1^ controls the steepness of the switching function for counting contacts. A boundary of *Q* = 0.5 is used to separate folded from unfolded states.

### φ-Value Calculation from MD Simulations.

To characterize folding mechanism, we used TPs from folding simulations for the *L* = 51 case at 291 K. Fifty independent simulations, each started from fully extended configurations, were carried out for 4 μs. The folding barriers for the *L* = 31 and 35 cases are very high at the same temperature; therefore, the TPs are obtained from unfolding simulations instead. Starting from native-like folded configurations, 50 unfolding simulations were carried out, with each trajectory being 4 μs long. TPs were defined as those portions of the simulation trajectory from the last time I27 samples the configuration with *Q* < 0.3 till the first time it samples a configuration with *Q* > 0.7 (in the folding direction; opposite for unfolding). ϕ-values were computed from the TPs using the approximation:

$$\phi \left(i\right)\approx \langle p\left(q_{ij}|TP\right)\rangle_{j:\left(i,j\right)\in native}.$$ [8]

In this equation, $p\left(q_{ij}|TP\right)$ is the probability that the native contact $q_{ij}$ between residues *i* and *j* is formed on TPs as defined above. We also characterized the importance of individual contacts in determining the folding mechanism using $p{\left(\text{TP|}q_{ij}\right)}_{\text{nn}}$, defined in Eq. **1** (i.e., the probability of being on a TP given that contact *q*_*ij*_ is formed and the protein is not yet folded). Having already calculated $p\left(q_{ij}|TP\right)$ above, evaluating $p{\left(TP|q_{ij}\right)}_{nn}$ required $p{\left(q_{ij}\right)}_{\text{nn}}$, the probability of a contact being formed in all nonnative fragments of the trajectory, and p(TP), the fraction of time spent on TPs. For *L* = 51, we obtained $p{\left(q_{ij}\right)}_{\text{nn}}$ directly from unbiased folding simulations, using the portion of the trajectory up to the first folding event (i.e., the first time *Q* > 0.7). For *L* = 31 or 35, where the protein is still relatively unstable, we determined it from unfolding simulations by computing $p\left(q_{ij}\right)$ separately for the unfolded and TP portions of the trajectory and combining them weighted by $p{\left(\text{TP}\right)}_{\text{nn}}$. We determined $p{\left(\text{TP}\right)}_{\text{nn}}$ via folding (*L* = 51 case) and unfolding (*L* = 31 and *L* = 35 cases) simulations (described above). For the *L* = 51 case, $p{\left(\text{TP}\right)}_{\text{nn}}=2t_{\text{TP}}/2t_{\text{TP}}+t_{\text{F}}^{\text{mfpt}}$, where $t_{\text{TP}}$ is the mean TP time and $t_{\text{F}}^{\text{mfpt}}$ is the mean first passage time for folding obtained from the maximum likelihood estimator $t_{F}^{mfpt}=\left[N_{fold}t_{fold}+\left(N-N_{fold}\right)t_{sim}\right]/N_{fold}$, where N is the total number of trajectories (N=50), $N_{fold}$ is the number of trajectories folding within 4 μs, $t_{fold}$ is the average folding time (of the trajectories which fold), and $t_{sim}$ is the length of the simulations (4 μs). For the *L* = 31 and *L* = 35 cases, it is less efficient to obtain the folding time $t_{\text{F}}^{\text{mfpt}}$ directly, and therefore we estimate it based on the mean first passage time for unfolding, $t_{\text{U}}^{\text{mfpt}}$, from unfolding simulations. $p{\left(\text{TP}\right)}_{\text{nn}}=2t_{\text{TP}}/2t_{\text{TP}}+\frac{p_{U}}{p_{F}}t_{\text{U}}^{\text{mfpt}}$, where $p_{U}$ and $p_{F}$ are the equilibrium populations of the unfolded and folded respectively determined from umbrella sampling.

## Supplementary Material

Supplementary File

Supplementary File

Supplementary File
