# Supplementary material for: Folding pathway of an Ig domain is conserved on and off the ribosome
Source: Proc Natl Acad Sci U S A. 2018 Nov 9;115(48):E11284–93. doi: 10.1073/pnas.1810523115 (PMC6275497; doi:10.1073/pnas.1810523115)
Supplement: Supplementary File [file pnas.1810523115.sapp.pdf]

Supporting Information for "The Folding Pathway of an Ig Domain is Conserved On and Off the Ribosome"

Pengfei Tian[1‡], Annette Steward[2‡], Renuka Kudva[3‡], Ting Su[5‡], Patrick J. Shilling[3], Adrian A. Nickson[2], Jeffrey J. Hollins[2], Roland Beckmann[5], Gunnar von Heijne[3,4#], Jane Clarke[2#] and Robert B. Best[1#]

[1]Laboratory of Chemical Physics, NIDDK, National Institutes of Health, 5 Memorial Drive, Bethesda, MD 20892-0520, USA.

[2]Department of Chemistry, University of Cambridge, Lensfield Road, Cambridge, CB2 1EW, UK.

[3]Department of Biochemistry and Biophysics, Stockholm University, SE-10691 Stockholm, Sweden.

[4]Science for Life Laboratory Stockholm University, Box 1031, SE-171 21 Solna, Sweden.

[5]Gene Center, Department of Biochemistry and Center for Integrated Protein Science Munich, CiPS-M, Feodor-Lynen-Strasse 25, Ludwig Maximilian University of Munich, 81377 Munich, Germany.

[‡]P.T., A.S., R.K. and T.S. contributed equally to this work.

[#]Corresponding authors

www.pnas.org/cgi/doi/10.1073/pnas.1810523115

Supplementary Figures

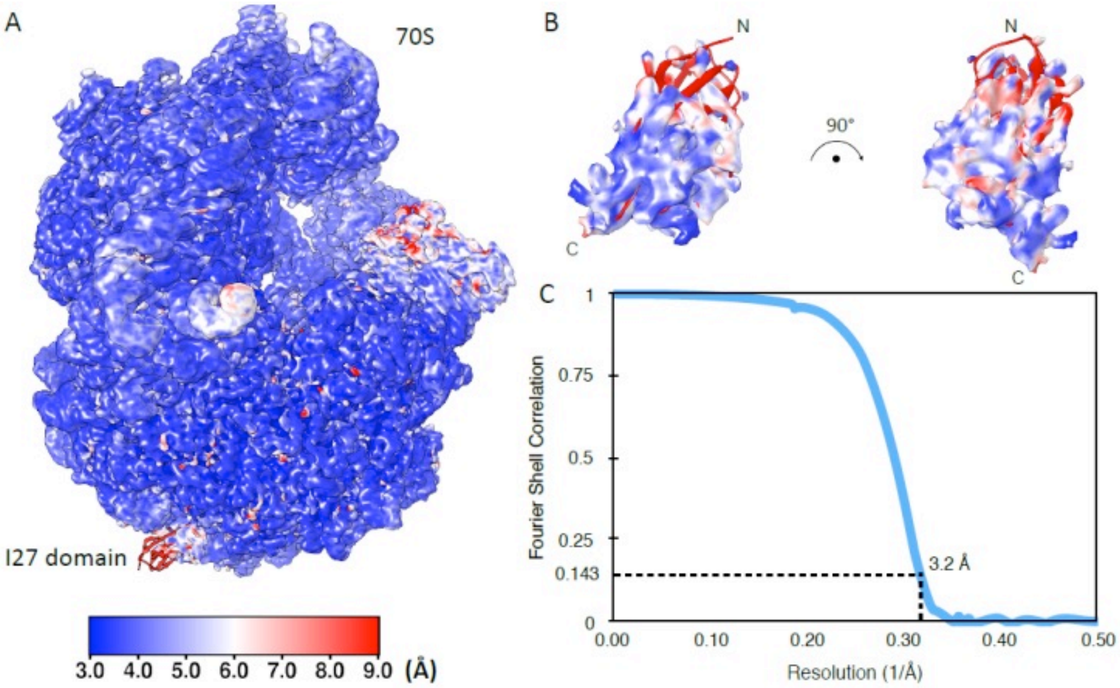

Figure S1. Resolution of the ribosome-nascent chain complex (RNC). (A) Calculation of the local resolution using Resmap (Kucukelbir, A. et al. Nat Methods 11, 63-65, 2014). The RNC density is displayed at 1.7 RMSD. (B) local resolution of the I27 domain. The I27 domain density is displayed at 2 RMSD. N and C termini are indicated. (C) Fourier-shell correlation (FSC) curve of the refined final map of the RNC, indicating the average resolution of 3.2 Å (at 0.143).

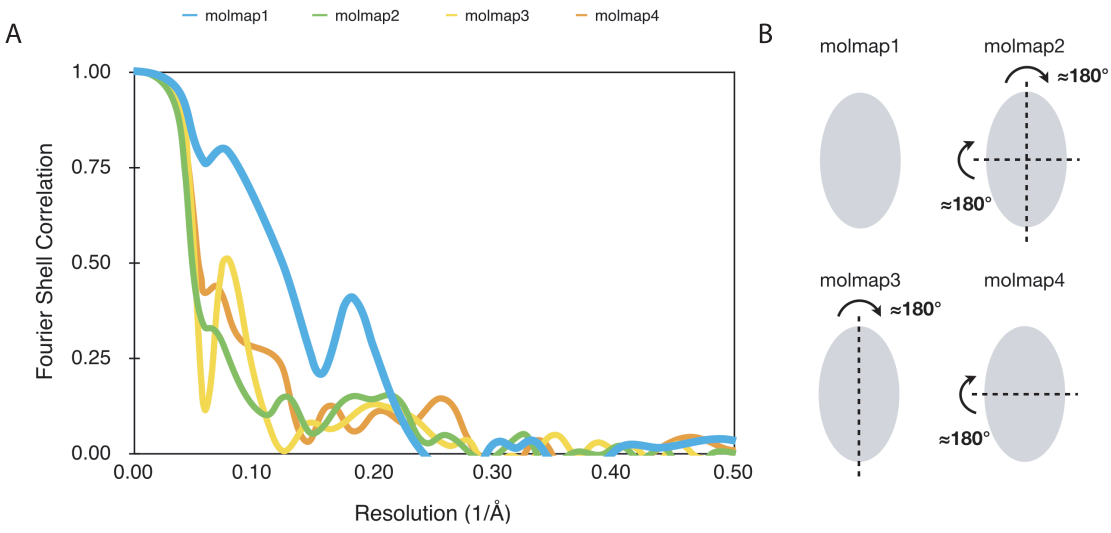

Figure S2. Validation of model orientation for I27 domain. To validate the orientation of the I27 domain model (PDB 1TIT) to its corresponding density, four possible orientations were tested. (A) The Fourier-shell correlations between the isolated I27 density and the map generated from the model of the final orientation (molmap1, blue) and the models fitted with the other three possibilities (molmap2, green; molmap3, yellow; molmap4, orange) were plotted. In the frequency range 0 to 0.2 (1000 to 5 Å) the correlation of molmap1 is significantly higher compared to all other orientation molmaps. (B) The illustration showing the relationship among the four model orientations. Since the density represents a flat ellipsoid, we used all four major and minor axes covering all possible orientations of the model fitting within the density.

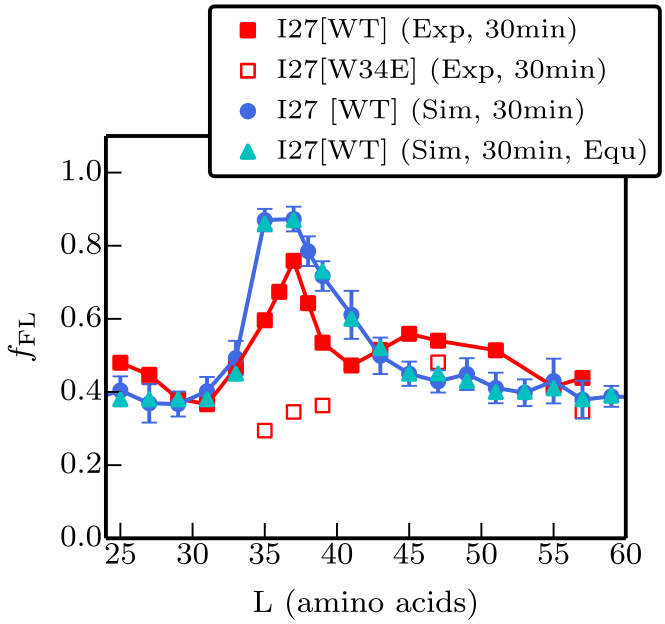

Figure S3. Experimental (red) and simulated (cyan) profiles of fraction full length protein, f_{FL}, obtained with a 30 min incubation. Note the higher background values compared to main text Figures 1 and 3D. Force profiles calculated from simulations using full kinetic scheme and pre-equilibrium model are shown in blue circles and cyan triangles respectively. The RMSD of the f_{FL} between experiment and simulation is 0.12.

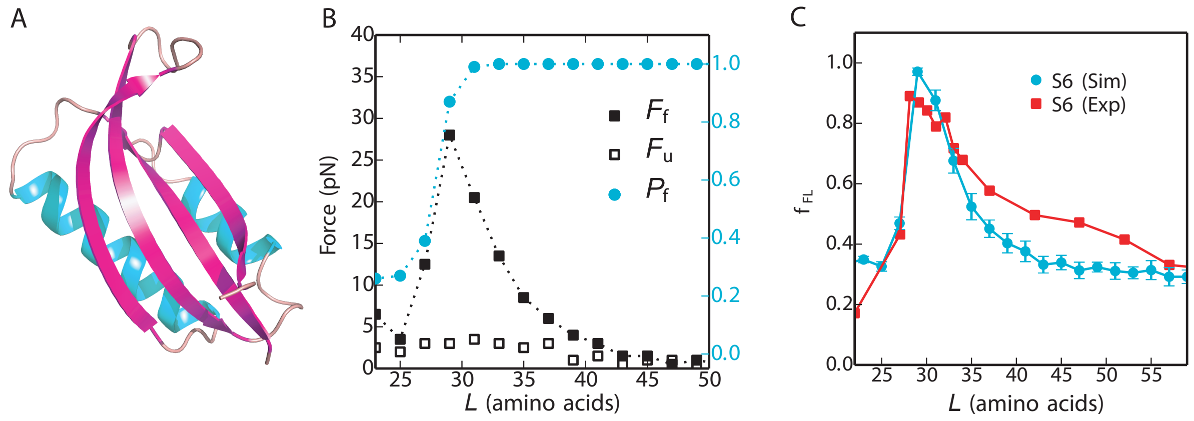

Figure S4. (A) Native structure of protein S6 (pdb code: 2KJV (84)) B) Average forces exerted on the AP by the unfolded state (F_f , filled black symbols) and folded state (F_u , empty black symbols) of S6 at different linker lengths *L*. The average fraction folded S6 for different *L*, P_f, is shown in cyan on the right axis. (C) Experimental (red) and simulated (cyan) force profiles for cotranslational folding of S6 based on pre-equilibrium kinetic solution.

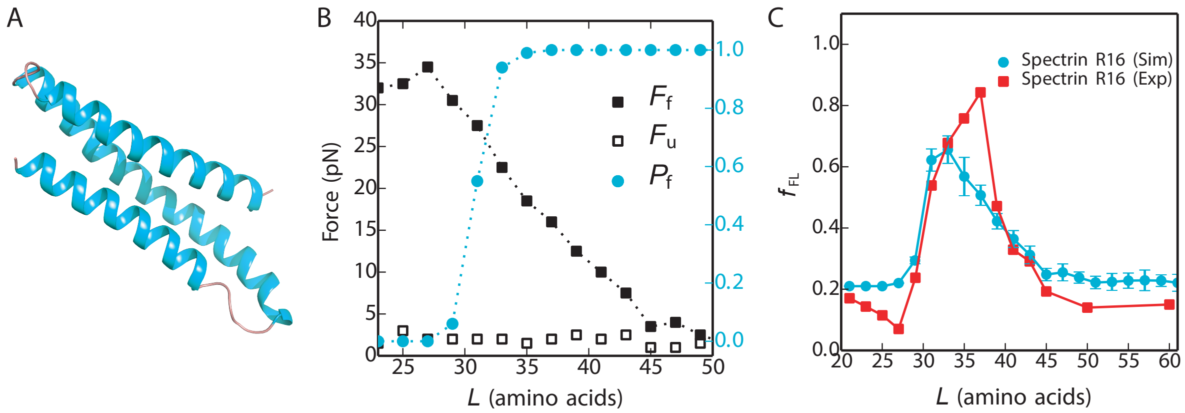

Figure S5. (A) Native structure of Spectrin R16 domain (PDB 1AJ3 (85)). (B) Average forces exerted on the AP by the unfolded state (F_u, empty black symbols) and folded state (F_f, filled black symbols) of R16 at different linker lengths L. The average fraction folded R16 for different L, P_f, is shown in cyan on the right axis. (C) Experimental (red) and simulated (cyan) force profiles for cotranslational folding of R16 based on the pre-equilibrium kinetic solution.

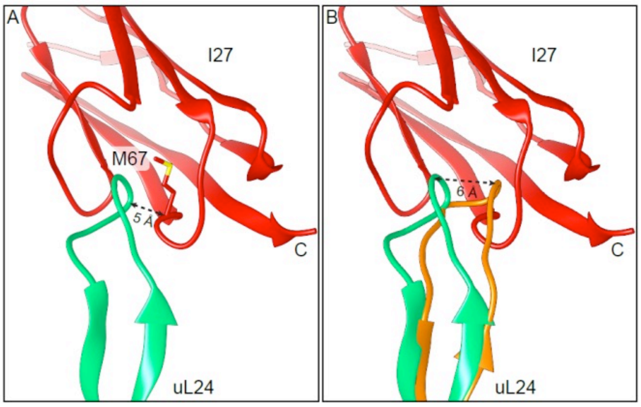

Figure S6. The I27 domain and a β hairpin in ribosomal protein uL24 close to the ribosomal exit tunnel. (A) Residue M67 in the I27 domain is located in close proximity to a β hairpin loop in uL24 in the cryo-EM structure of I27-TnaC[L=35] RNCs. (B) The uL24 β hairpin in the I27-RNC (light green; re-modeled based on PDB 5NWY) is ~ 6 Å shifted (distance measured via the backbone of Pro50) compared to its location in the VemP-RNC (orange; PDB 5NWY) and the TnaC-RNC (PDB 4UY8, not shown for clarity). C represents the C terminus of the I27 domain. The loop tip residues V49-N53 of the uL24 β hairpin are in contact with I27. Residues are defined as being in contact if any pair of heavy atoms, one from each residue, are closer than 4.5 Å.

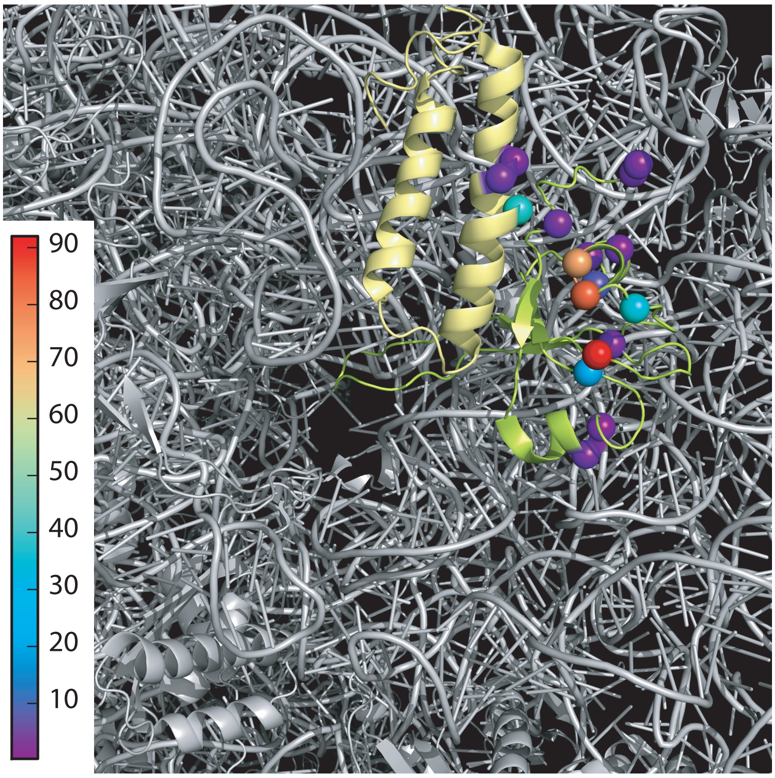

Figure S7. The probability of individual hydrophobic residues on the ribosome surface (from proteins uL23 and uL29) being in contact with I27[M67A], from the reweighted ensemble obtained from umbrella sampling simulations at a linker length of L=45. uL29 and uL23 are coloured in yellow and green respectively. The CA atoms of the top ranking residues are shown as beads coloured according to the contact probability (given as a percentage in the colour bar). A ribosome residue is defined as being in contact with I27[M67A] if it is within 8.0Å of any residues of I27[M67A] that are involved in the attractive interactions (Eq. 3).

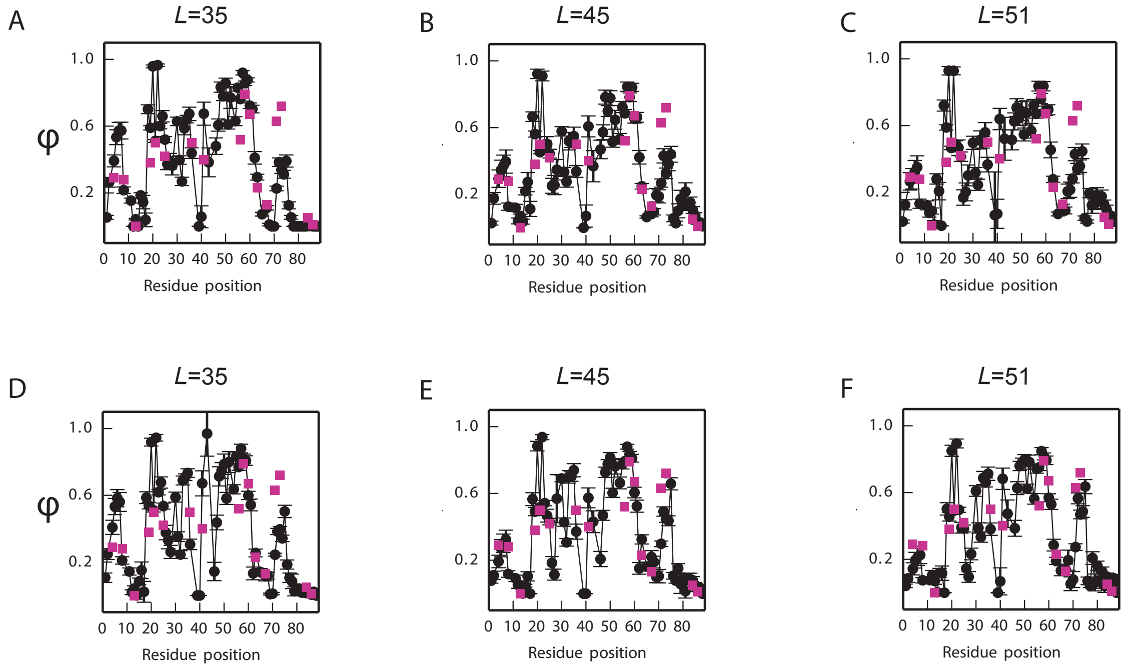

Figure S8. Simulated folding pathways for ribosome-tethered I27 [M67A]. LH column, *L*=35; middle column, *L*= 45; RH column, *L*=51. Simulated ϕ-values for I27[M67A] (black) are shown without (A-C) and with (D-F) hydrophobic attractions between ribosome and I27[M67A]. ϕ-values determined by *in vitro* folding of purified I27 WT are shown as red squares.

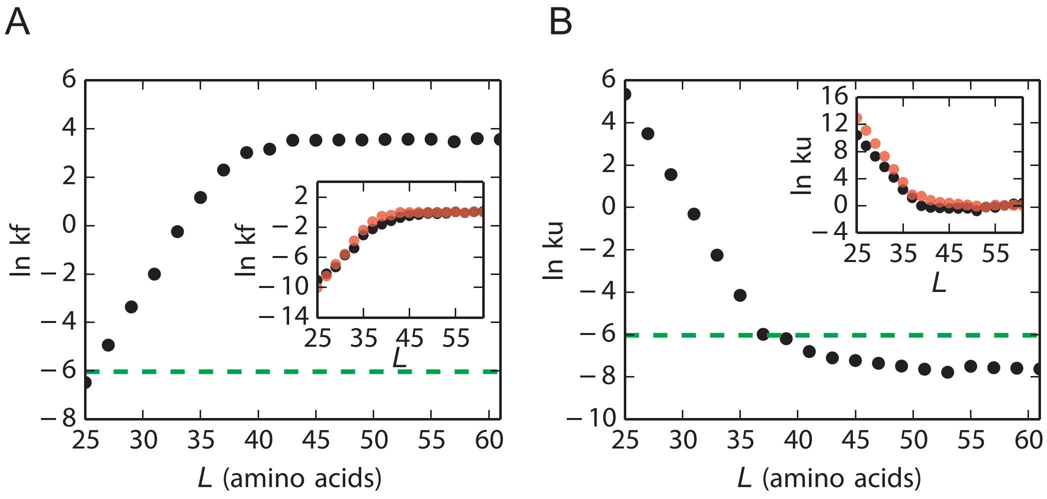

Figure S9. Dependence of folding rate k_f (left) and unfolding rate k_u (right) on the length of the linker between the AP and I27[WT]. Rates determined directly from simulations have been scaled so that k_f and k_u at large linker lengths are equal to the experimental values determined for the isolated protein. The green dashed line indicate the force-dependent escape rate of ~ 2.4 x 10^{-3} s^{-1} obtained at the force of 20 pN. (insets) The linker length dependent folding/unfolding rates of the mutant I27[L58A] (in red) are overlaid with the rates for the wild type (black). Folding and unfolding rates are divided by the values at the longest linker length (L=61). Here, the mutant I27[L58A] is modelled explicitly by weakening all native contacts to residue 58 by the same factor (40%), with which the loss of folded state stability (3.21 kcal/mol) was comparable to experiment (3.23±0.1 kcal/mol).

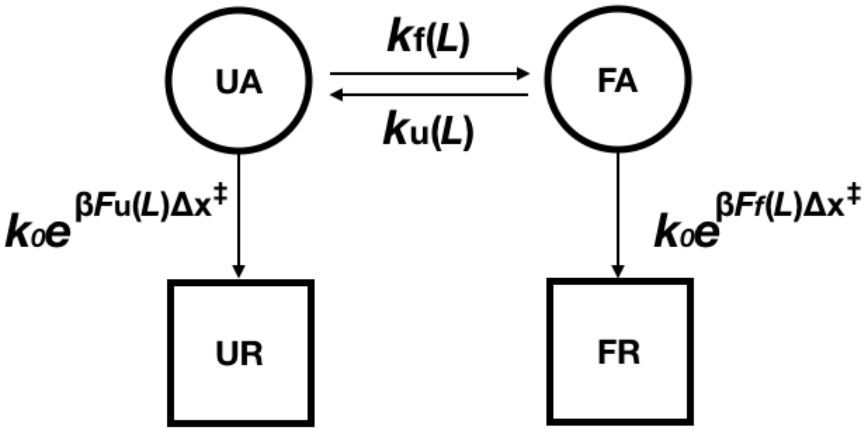

Figure S10. Schematic for the full kinetic model which describes the escape pathway of I27 from the ribosome. k_f and k_u are the linker length-dependent folding and unfolding rates respectively. UA: I27 is unfolded and arrested by ribosome. FA: I27 is folded and arrested by ribosome. UR: I27 is unfolded and released from ribosome. FR: I27 is folded and released from ribosome.

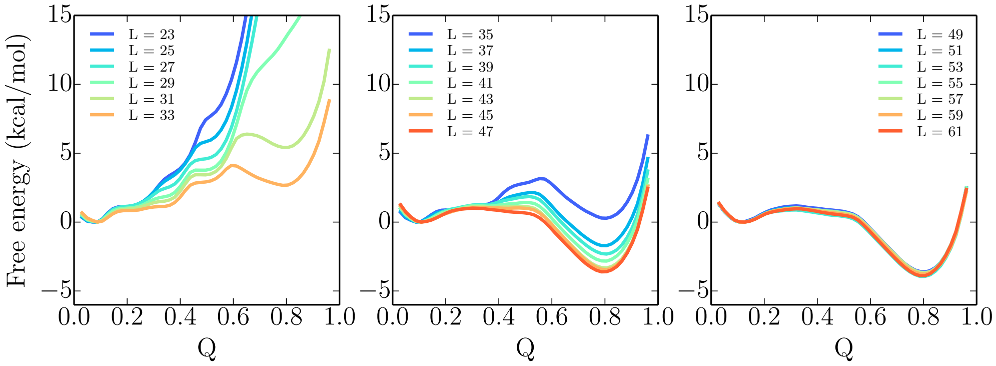

Figure S11. Simulation free energy $F(Q)$ projected on the fraction of native contacts, Q, for I27 folding with different linker lengths (as indicated in legend) at 291K.

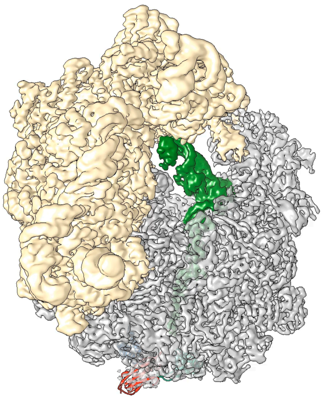

Supporting Video S1. Cryo-EM density of ribosome and I27 (one static frame of the video). Video showing cryo-EM map for I27[L=35] RNCs. 30S in yellow, 50S and I27 domain in grey, tRNA and nascent chain in green, the model (PDB 1TIT) of I27 domain in red.

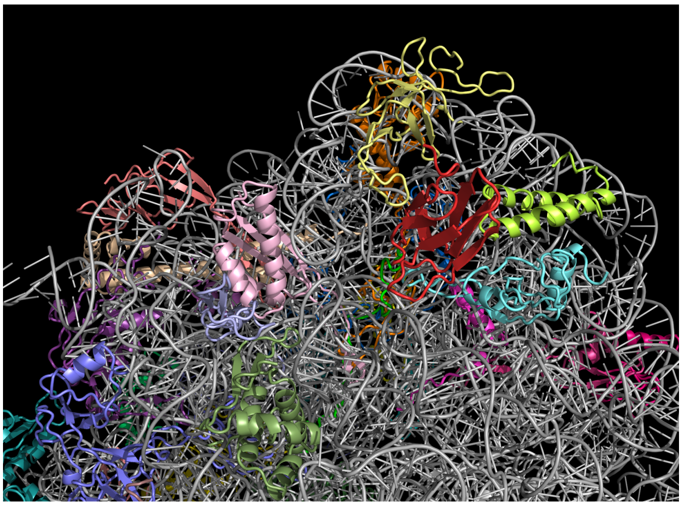

Supporting Video S2. MD folding simulation (one static frame of the video). Video showing an unbiased 1.8 μsec fragment of an MD trajectory of I27 folding and unfolding at linker length *L*=35. Ribosomal 23s rRNA is shown in white cartoon mode, ribosomal proteins uL24, uL29, uL23 are shown in yellow, lime and cyan respectively. I27 and linker are in red and green cartoon mode respectively.
